# Promotion of Peripheral Nerve Regeneration by Stimulation of the Extracellular Signal‐Regulated Kinase (ERK) Pathway

**DOI:** 10.1002/ar.24126

**Published:** 2019-04-22

**Authors:** Barbara Hausott, Lars Klimaschewski

**Affiliations:** ^1^ Department of Anatomy, Histology and Embryology, Division of Neuroanatomy Medical University Innsbruck Innsbruck Austria

**Keywords:** growth factor, RTK, neuronal survival, axon, Sprouty

## Abstract

Peripherally projecting neurons undergo significant morphological changes during development and regeneration. This neuroplasticity is controlled by growth factors, which bind specific membrane bound kinase receptors that in turn activate two major intracellular signal transduction cascades. Besides the PI3 kinase/AKT pathway, activated extracellular signal‐regulated kinase (ERK) plays a key role in regulating the mode and speed of peripheral axon outgrowth in the adult stage. Cell culture studies and animal models revealed that ERK signaling is mainly involved in elongative axon growth *in vitro* and long‐distance nerve regeneration *in vivo*. Here, we review ERK dependent morphological plasticity in adult peripheral neurons and evaluate the therapeutic potential of interfering with regulators of ERK signaling to promote nerve regeneration. Anat Rec, 302:1261–1267, 2019. © 2019 Wiley Periodicals, Inc.

## INTRODUCTION

In response to nerve injury, receptor tyrosine kinases (RTKs) are activated by growth factors, thereby inducing several intracellular signaling processes that lead the way to successful regeneration mainly via protein kinase B (PKB/AKT) and extracellular signal‐regulated kinase (ERK) dependent pathways. Activation of phosphatidylinositol‐3‐kinase (PI3K) and downstream target AKT is absolutely required for axon regeneration in the adult peripheral nervous system, since inhibition of PI3K limits spontaneous as well as growth factor‐induced axon outgrowth (Klimaschewski et al., 2013). Phosphorylation of AKT is elevated in lesioned peripheral axons, which is required for induction of outgrowth and branching. Molecular analysis revealed that the PI3K/AKT pathway is crucially involved in the reorganization of microtubules via inhibition of GSK‐3β and of actin filaments through the regulation of small GTPases (Rac and Cdc42). The functional significance of PTEN/PI3K/Rho/ROCK dependent signaling in peripheral nerve regeneration and possible interactions between the Rho/ROCK and the ERK pathway have been described (Auer et al., 2011; Hensel et al., 2015; Krishnan et al., 2016).

**Figure 1:**
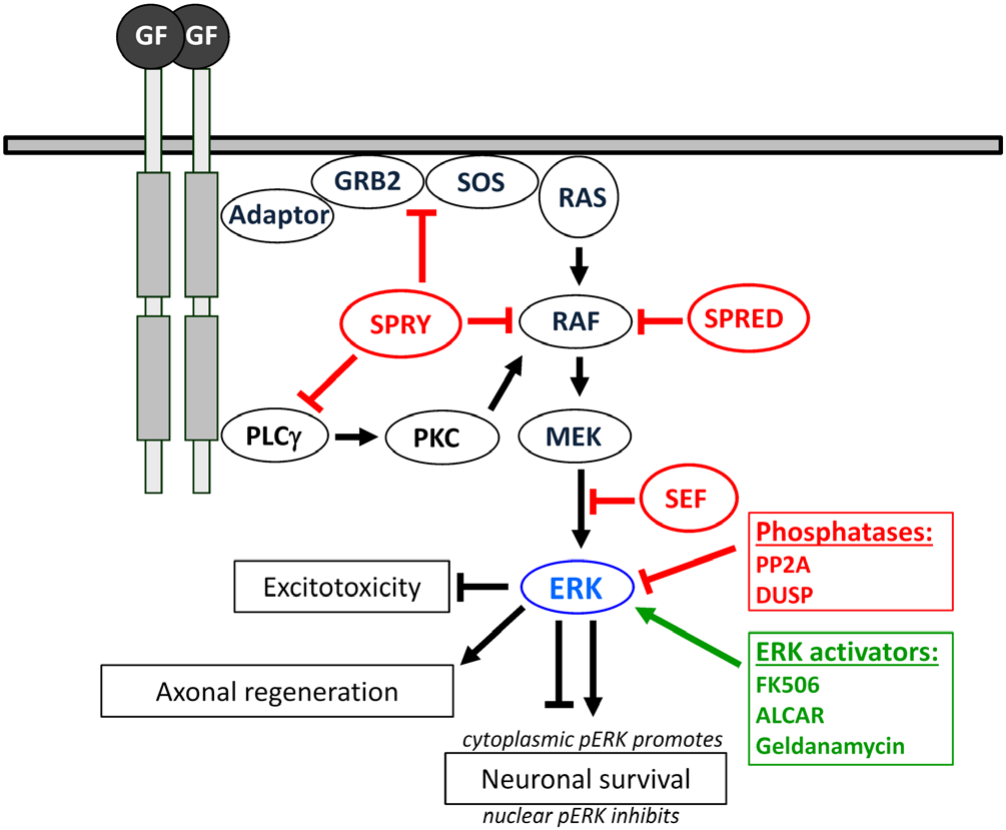
The RAS/RAF/MEK/ERK pathway: Upon growth factor (GF) activation RTKs auto‐phosphorylate and recruit the SH2 domain‐containing GRB2, which interacts with the guanine nucleotide exchange factor SOS that induces release of GDP from RAS, which subsequently binds GTP. RAS recruits RAF to the membrane. Active RAF then acts on MEK dual specificity kinase that phosphorylates ERK on both threonine and tyrosine residues. The dimerized form of ERK exerts a variety of post‐transcriptional but also nuclear effects by phosphorylation of transcription factors. The RAS/ERK pathway is tightly regulated. Several phosphatases including PP2A and DUSPs inactivate ERK in the cytoplasm or nucleus. Furthermore, endogenous modulators such as SPRY, SPRED, or SEF inhibit ERK signaling upstream and downstream of RAS. Activating drugs such as FK506, ALCAR, or Geldanamycin enhance ERK signaling. Increased pERK levels promote peripheral axon regeneration and neuronal survival but inhibit excitotoxicity. Downregulation of ERK inhibitors such as SPRY2 or treatment with ERK activators like FK506 has been demonstrated to promote peripheral nerve regeneration as well.

The rat sarcoma (RAS)‐, rapidly accelerated fibrosarcoma (RAF)‐, mitogen‐activated and extracellular signal‐regulated kinase (MEK) pathway, also known as the mitogen‐activated protein kinase or RAS/ERK pathway, is activated by specific ligands followed by auto‐phosphorylation of tyrosine residues at the cytoplasmic tails of RTKs (Fig. 1). This in turn leads to the recruitment of adapters such as growth factor receptor‐bound protein 2 (GRB2) and guanine nucleotide exchange factor son of sevenless (SOS). The interaction of SOS with the small GTPase RAS results in GTP loading and activation of RAS. RAS recruits RAF to the plasma membrane, which activates MEK, a dual specificity kinase that phosphorylates ERK on both threonine and tyrosine. Consequently, activated ERK affects several target proteins located in the cyto‐ and axoplasm and will be retrogradely transported to the neuronal cell body. The dimerized form of ERK actively translocates into the nucleus and phosphorylates a variety of transcription factors such as Elk1, c‐Fos, or c‐Jun (Avruch, 2007).

Starting in the 1980s, the ERK pathway provided the basis for describing how extracellular growth factors propagate intracellular signals that control the morphological and biochemical fate of nearly all cells. In the nervous system, ERK activation was shown to regulate neuronal survival and axon growth by influencing axonal transport, local protein synthesis, and gene expression. Moreover, ERK dependent post‐transcriptional mechanisms modify axon assembly via changes in polymerization of microtubules and actin filaments (Zhou and Snider, 2006). Thus, it is not surprising that inhibition of ERK induces actin depolymerization and growth cone collapse (Atwal et al., 2000; Goold and Gordon‐Weeks, 2005). Here, we will focus on the ERK pathway in adult peripheral axon outgrowth and provide an outlook on potential new strategies to improve the outcome of nerve lesions.

Active ERK has also been shown to play a crucial role during development of the peripheral nervous system (Newbern, 2015). Although early embryonic phases of neuronal differentiation and axon growth are mainly independent of ERK, it is critical for nociceptive innervation of cutaneous target fields at late embryonic and early postnatal stages. ERK signaling is particularly relevant in Schwann cells during early peripheral nervous system (PNS) development and myelination. Peripheral nerves of ERK knockout mice are devoid of Schwann cell progenitors, which cause nerve defasciculation, loss of all peripheral projections, and neuronal cell death. Deletion of ERK in Schwann cell precursors disrupts differentiation and induces hypomyelination of axons (Newbern et al., 2011). The reactivation of ERK in adult myelinating Schwann cells induces reversion to a Schwann cell progenitor‐like state (Napoli et al., 2012) supporting the important role of ERK in Schwann cell differentiation. Schwann cells with sustained ERK activation hypermyelinate PNS axons during development, and myelin thickness is disproportionately increased relative to the axonal diameter (Ishii et al., 2013).

### Regulation of the RAS/ERK Pathway

It is evident that an important signaling pathway needs tight regulation (Fig. 1; Kolch, 2005). Protein phosphatase 2A PP2A) and dual specificity protein phosphatases (DUSPs) inhibit ERK signaling by dephosporylation (Ramos, 2008). Furthermore, Sprouty (SPRY), Sprouty‐related EVH1 domain‐containing protein (SPRED), or similar expression to FGF (SEF) proteins belong to a group of endogenous modulators of ERK activity (Bundschu et al., 2007; Edwin et al., 2009; Mason et al., 2006; Ron et al., 2008; Torii et al., 2004b). Recently, we reviewed the possible significance of SPRY proteins as signaling integrators in the nervous system under normal and pathological conditions (Hausott and Klimaschewski, 2018).

Four functionally conserved SPRY proteins exist in mammals (Ozaki et al., 2005). In response to RTK activation, SPRY1 and ‐2 bind GRB2 thereby preventing RAS/ERK activation (Hanafusa et al., 2002; Tefft et al., 2002). Furthermore, SPRY2 and ‐4 inhibit ERK activation downstream of RAS by binding RAF (Sasaki et al., 2003; Yusoff et al., 2002). Thus, SPRY2 interferes with the RAS/ERK pathway upstream and downstream of RAS. We found that SPRY2 is highly expressed in the PNS and downregulation of SPRY2 promotes axon regeneration *in vitro* and *in vivo* (Hausott et al., 2009; Marvaldi et al., 2015).

SPREDs inhibit ERK activation by suppressing the activation of RAF. Overexpression of SPRED prevents nerve growth factor (NGF) induced differentiation of PC12 cells, whereas antibodies against SPRED augment neurite outgrowth of PC12 cells treated with low NGF concentrations (Wakioka et al., 2001). SPRED is expressed in the zebrafish brain and its downregulation induces cell proliferation in the adult fish brain (Lim et al., 2016). However, data about the role of SPREDs in peripheral nerve regeneration are lacking.

SEF was first identified as an inhibitor of RAS‐mediated fibroblast growth factor (FGF) signaling in the zebrafish (Furthauer et al., 2002; Tsang et al., 2002). It prevents nuclear translocation of ERK by inhibition of the dissociation of the ERK/MEK complex. Knockdown of SEF results in the nuclear accumulation of ERK and activation of Elk1 (Torii et al., 2004a). ERK retained in the cytoplasm is unable to promote neurite extension in PC12 cells, whereas its nuclear targeting results in neuronal differentiation (Robinson et al., 1998). Thus, overexpression of Sef inhibits FGF2‐ and NGF‐induced neurite outgrowth by PC12 cells (Xiong et al., 2003). Sef is expressed in the spinal cord and in dorsal root ganglia (DRG) and upregulated in response to a sciatic nerve crush at the lesion site (Grothe et al., 2008).

### Relevance of ERK for Neuronal Survival

Activation of ERK signaling protects different cell types and Schwann cell precursors against apoptosis but its role for promoting the survival of neurons is controversially discussed. The primary survival pathway in adult neurons is mediated via PI3K/AKT signaling described above (Crowder and Freeman, 1998; Dudek et al., 1997). Although activation of ERK moderately stimulates survival signaling in sympathetic neuron cultures, MEK inhibitors exert no dramatic effects on NGF‐dependent survival (Mazzoni et al., 1999; Virdee and Tolkovsky, 1996). In line with these *in vitro* results, deletion of B‐RAF, which reduces phosphorylation of ERK, does not lead to cell death of DRG neurons (Zhong et al., 2007). Thus, ERK signaling is not a major mediator of neuronal survival during development although ERK is required for postnatal survival of nociceptive sensory neurons (O'Brien et al., 2015). However, this survival effect is likely due to impaired axon growth that reduces access to target derived growth factors that promote survival.

The major role of the ERK pathway in neuronal survival appears to relate to the neuronal response to toxicity, for example, ERK is activated by stress to counteract apoptosis in cortical neurons (Hetman et al., 1999). Similarly, MEK protects sympathetic neurons against apoptosis induced by cytosine arabinoside and retinal ganglion cells from death following axotomy (Anderson and Tolkovsky, 1999; Shen et al., 1999). However, inhibition of ERK had no effect on neuronal survival in the facial nerve lesion model, although axotomy of the facial nerve increased ERK phosphorylation in the facial brainstem nucleus 7 days after injury (Huang et al., 2017). Similar effects were observed in response to a sciatic nerve crush (Agthong et al., 2009) suggesting that ERK does not play a major role in neuronal survival after peripheral axotomy. Interestingly, several studies even suggested a role for ERK in promoting neuronal and glial cell death in the brain (Subramaniam and Unsicker, 2010). For apoptosis to occur, ERK appears to require nuclear translocation, whereas sustained ERK activation in the cytoplasm results in neuronal survival (Stanciu and DeFranco, 2002; Subramaniam et al., 2004).

### Role of ERK in Nerve Regeneration

Peripheral nerves are provided with the ability to regenerate in response to injury but the rate of regeneration at 1–3 mm per day is slow and functional outcomes are often poor in patients. The regenerative capacity of axons and the growth support of Schwann cells decline with time and distance from injury (Fu and Gordon, 1995). Hence, regenerating peripheral axons require substantial growth support to achieve a successful functional outcome. CNS neurons are refractory to axon regeneration due to a plethora of inhibitory molecules in myelin and in the extracellular matrix. However, just the removal of inhibitory molecules has not proven to enable long‐distance axon growth. Thus, neuron intrinsic pathways that promote axon regeneration are of special interest to find new strategies that improve functional recovery after axonal injury.

Early studies suggested an important role of the RAS/ERK pathway for axon growth. Cell culture studies with embryonic DRG and superior cervical ganglion neurons revealed that ERK is strongly involved in axonal elongation (Atwal et al., 2000; Markus et al., 2002). In adult DRG neurons, basic fibroblast growth factor (FGF2) treatment induces prominent ERK phosphorylation and significantly improves elongative over branching axon outgrowth of adult sensory neurons in response to a preconditioning sciatic nerve lesion (Hausott et al., 2009; Klimaschewski et al., 2004). The effects on neurite outgrowth seem to be dependent on the duration and/or strength of the ERK signal not unlike PC12 pheochromocytoma cells, which exhibit sustained ERK activation before neurite outgrowth is observed (Traverse et al., 1992).

Other studies demonstrated that in dissociated adult DRG neuron cultures ERK inhibition has no effect on intrinsic axon outgrowth (Kimpinski and Mearow, 2001; Tucker et al., 2008) or may even stimulate process formation (Jones et al., 2003). Furthermore, regenerative axon growth in response to a preconditioning lesion was not affected by ERK inhibition in dissociated DRG cultures (Liu and Snider, 2001). Similarly, ERK blockade did not interfere with spontaneous axon outgrowth of adult DRG explants, while growth factor‐induced outgrowth was significantly impaired (Sjogreen et al., 2000; Sondell et al., 1999). Nevertheless, ERK is clearly required for axotomy‐induced growth cone formation after lesion and for axon growth by preaxotomized DRG explants (Chierzi et al., 2005; Wiklund et al., 2002).

Phosphorylation of ERK is enhanced in regenerating nerves in response to sciatic nerve crush and transection (Agthong et al., 2006; Sheu et al., 2000; Yamazaki et al., 2009). Active ERK is transported retrogradely to the neuronal nucleus via vimentin along axons and activates transcription factors such as Elk1 that induces the regenerative response (Perlson et al., 2005). The vimentin/ERK complex protects ERK from dephosphorylation enabling long distance transport of phosphorylated pERK within the cell (Perlson et al., 2006). Since the interaction of pERK and vimentin is calcium dependent, this signal may provide information on the injury and on the degree of damage. Inhibition of ERK clearly impairs sciatic nerve regeneration (Agthong et al., 2009). Likewise, blockade of ERK phosphorylation significantly reduces the length of regenerated axons in the facial nerve lesion model (Huang et al., 2017).

As discussed above, SPRY2 is one of the major inhibitors of RAS/ERK activation and expressed at high levels in adult DRG neurons (Hausott et al., 2009). Downregulation of SPRY2 enhances phosphorylation of ERK and promotes axon regeneration *in vitro* and *in vivo* (Hausott et al., 2009; Marvaldi et al., 2015). Conversely, overexpression of SPRY2 inhibits axon growth by adult DRG neurons. Although SPRY2 mRNA is not regulated in response to a sciatic nerve lesion (Hausott et al., 2009), SPRY2 protein levels are reduced post‐transcriptionally by microRNA miR‐21 (Strickland et al., 2011). Upregulation of miRNA‐21 is observed 2 days after axotomy, and this increase is sustained up to 28 days after injury indicating an important role of SPRY2 during peripheral axon regeneration. In fact, heterozygous SPRY2 knockout mice reveal faster motor recovery, improvement in behavioral motor tests, and higher numbers of myelinated fibers in the regenerating sciatic nerve. Furthermore, increased levels of GAP‐43 mRNA are observed in nerves of heterozygous SPRY2 knockout mice in response to a sciatic nerve crush (Marvaldi et al., 2015). This downstream target of ERK signaling supports elongative axon growth that is required for successful regeneration (Donnelly et al., 2013).

DRG neurons provide a useful model system to study regenerative properties because they have a central branch with limited regenerative capacity and a peripheral branch that regrows after lesion. Although ERK is activated in response to dorsal root injury, the regenerative capacity is poor because of the strong effect of inhibitory injury signals such as ROCK, among others (Mar et al., 2016). Interestingly, activation of B‐RAF enables reinnervation of the adult dorsal horn after dorsal root crush injury (O'Donovan et al., 2014) demonstrating positive effects of enhanced ERK signaling on axon regeneration in the presence of growth inhibitory extracellular signals.

Schwann cells play a crucial role during PNS regeneration as well (Gordon, 2014). In response to injury, they de‐differentiate and proliferate, which contributes to the removal of axonal and myelin debris and the secretion of factors that are supportive for the growth of regenerating nerve fibers. Subsequently, Schwann cells re‐differentiate to generate myelin‐forming and non‐myelin‐forming (Remak) cells. Acute strong ERK activation in Schwann cells in the absence of axonal injury is sufficient to induce demyelination and recruitment of inflammatory cells in the adult nerve and remyelination occurs as the phosphorylated ERK levels normalize again (Napoli et al., 2012). Thus, the strength and temporal profile of ERK induction appears to be a key factor in the regulation of Schwann cell function, which is supported by recent studies demonstrating that moderate ERK induction in Schwann cells had no effect on myelin stability in the intact adult nerve. In response to a sciatic nerve crush, however, moderate ERK induction increases the inflammatory response and accelerates the clearance of the myelin debris, but at later stages of regeneration functional recovery is delayed due to morphological defects in myelinated and non‐myelinated fibers. Decreased myelin stability and reduced internodal length are observed without changes in myelin thickness, whereas in unmyelinated fibers Remak bundle formation is disrupted (Cervellini et al., 2018). In contrast, another recent study demonstrates that ERK activation induced by hepatocyte growth factor improves remyelination through enhanced Schwann cell migration after a sciatic nerve crush (Ko et al., 2018). Taken together, these results emphasize the importance of the strength, duration, and location of ERK activation during PNS regeneration.

### Interference with ERK in Lesioned Neurons

Several clinical trials using neurotrophic factors as treatments for PNS disorders unfortunately failed (Thoenen and Sendtner, 2002). The method and site of factor administration as well as strong PI3K/AKT mediated stimulation of axon branching turned out to be major problematic factors. Thus, direct activation of specific signaling pathways that enhance long‐distance axon elongation may provide a better strategy to promote nerve regeneration.

As outlined in our recent review (Hausott and Klimaschewski, 2018), SPRY2 acts as major inhibitor of the ERK pathway and its downregulation promotes peripheral nerve regeneration (Marvaldi et al., 2015). The effects of other endogenous ERK modulators such as SEF or SPRED in peripheral nerve regeneration require further investigation. Besides SPRY2 and ‐4, they may provide suitable targets for interference as well as phosphatases that inactivate RAF, MEK, or ERK such as PP2A or DUSP6. DUSP6 appears to be particularly promising since it is increased by NGF in a negative feedback loop (Finelli et al., 2013).

Gene therapy provides a useful tool for specific downregulation or knockout of neuronal targets, since efficient siRNA treatments and viral gene transfer of shRNAs are now available for humans. Gene replacement therapy has been demonstrated to promote survival of patients with spinal muscular atrophy following a single intravenous infusion of adeno‐associated virus containing DNA coding for SMN1 (Mendell et al., 2017).

Pharmacological strategies to enhance ERK signaling include drugs like Tacrolimus (FK506), Geldanamycin, or ALCAR (acetyl‐l‐carnitine, Chan et al., 2014). FK506 acts as immunosuppressant that improves axon growth through enhanced ERK activation and increased levels of GAP‐43 (Gold et al., 1998; Gold and Zhong, 2004). Local administration of FK506 to PNS injuries avoids the side effects of systemic immunosuppression and improves muscle mass and motor recovery in animal models (Azizi et al., 2012; Mekaj et al., 2017). The antibiotic Geldanamycin promotes regeneration in nerve crush and transections models (Sun et al., 2012). The rate of regenerating axons is comparable to that of FK‐506 but the functional recovery does not match the performance of FK506 due to secondary effects of FK506 on Wallerian degeneration. However, only few studies about the function of Geldanamycin in nerve regeneration are available and the poor water solubility and potential for hepatotoxicity limit its application in humans (Chan et al., 2014). By contrast, ALCAR is an antioxidant with proven drug safety that can be administered orally. Several studies supported the usefulness of ALCAR in sciatic nerve lesion models after local or systemic treatment (Mohammadi and Amini, 2017; Wilson et al., 2010). Its high drug safety makes it a potential therapeutic agent that has already been tested in humans to enhance regeneration in chronic diabetic neuropathy (Sima et al., 2005). However, all of these drugs are not specific for ERK but activate several other pathways (Chan et al., 2014). Therefore, further testing of ERK activating drugs that enhance PNS regeneration and have low side effects is warranted to obtain effective treatments for patients with peripheral nerve injuries in the future.
